# A Clinical Rationale for Assessing the Impact of Childhood Sexual Abuse on Adjunctive Subcutaneous Esketamine for Treatment-Resistant Depression

**DOI:** 10.3389/fpsyt.2021.608499

**Published:** 2021-08-17

**Authors:** Eduardo Jorge Muniz Magalhães, Luciana Maria Sarin, Lorena Catarina Del Sant, Ana Cecília Lucchese, Carolina Nakahira, Marco Aurélio Tuena, Camila Brito Puertas, Victor Augusto Rodovalho Fava, Rodrigo Simonini Delfino, Juliana Surjan, Matheus Souza Steglich, Matheus Ghossain Barbosa, Guilherme Abdo, José Alberto Del Porto, Charles B. Nemeroff, Hugo Cogo-Moreira, Acioly Luiz Tavares Lacerda, Andrea Feijo Mello

**Affiliations:** ^1^Department of Psychiatry, Federal University of São Paulo, São Paulo, Brazil; ^2^Program for Care on Affective Disorders, Department of Psychiatry, Federal University of São Paulo, São Paulo, Brazil; ^3^Department of Psychiatry and Behavioral Sciences, Institute of Early Life Adversity Research, Dell Medical School, University of Texas at Austin, Austin, TX, United States; ^4^Department of Education, ICT and Learning, Faculty of Teacher Education and Languages, Østfold University College, Halden, Norway; ^5^Laboratory of Integrative Neuroscience, Department of Psychiatry, Federal University of São Paulo, São Paulo, Brazil; ^6^CNS Unit, BR Trials, São Paulo, Brazil; ^7^Program for Research and Care on Violence and Post-traumatic Stress Disorder, Department of Psychiatry, Federal University of São Paulo, São Paulo, Brazil

**Keywords:** child sex abuse, treatment-resistant depression, esketamine, sex, directed acyclic graph

## Abstract

**Background:** A history of child sexual abuse (CSA) is related to higher suicide rates and poor treatment outcomes in depressed adult patients. Twenty years after the first study investigating the effects of ketamine/esketamine on depression and suicide, there is a lack of data on the CSA effects on this emerging treatment. Here, we assess the impact of CSA on adjunctive subcutaneous (SC) esketamine for treatment-resistant depression (TRD).

**Methods:** A directed acyclic graphic (DAG) was designed to identify clinical confounders between CSA and esketamine predictors of response. The confounders were applied in a statistical model to predict depression symptom trajectory in a sample of 67 TRD outpatients.

**Results:** The patient sample had a relatively high prevalence rate of CSA (35.82%). Positive family history of first-degree relatives with alcohol use disorder and sex were clinical mediators of the effects of esketamine in a CSA adult population. Overall, the presence of at least one CSA event was unrelated to esketamine symptom reduction.

**Conclusions:** Unlike responses to conventional antidepressants and psychotherapy, CSA does not appear to predict poor response to esketamine.

## Introduction

The adverse effects of childhood abuse on health are substantial and well-documented. The WHO estimated in 2002 that 150 million girls and 73 million boys under the age of 18 had suffered various forms of sexual violence (1). Females have a two- or threefold higher risk compared with males to be sexually abused during childhood (2), resulting in a worldwide prevalence of 15–19.7% for women and 7.6–8% for men (3). Regrettably, most studies solely include reports from children's protection services, detecting only a small fraction of cases (4). This type of trauma can be considered as “toxic” and causes a prolonged activation of the body's stress response system (5). It impairs and affects regulation, impulse control, sense of self, socialization (6), and the brain changes have enduring consequences throughout life, mediating a negative trajectory to mood disorders in this population (7).

Depression is a disorder long known to be associated with CSA (8); poorer outcomes are related to CSA severity (9). Meta-analytic reviews have found evidence of associations between CSA and adult depression (10, 11). Despite the significant correlations between childhood abuse and adult depression, little is known about its specific effects on treatment (12), although, it is associated with reduced responsiveness to antidepressant pharmacotherapy and psychotherapy (13).

In view of the need for more effective treatments for individuals with TRD and CSA history, we sought to determine whether a subpopulation of patients with CSA and a current TRD episode would benefit from an investigational protocol with multiple subcutaneous (SC) esketamine injections. The subcutaneous route of administration is a reasonable alternative that leads to similar plasma concentrations and more feasible procedure for many clinics (14). A recent study outlined the cardiovascular safety of multiple SC esketamine injections in TRD (15).

To answer our research question, the relationship between exposure and outcome can be clarified using information from previous studies on CSA and predictors of response to ketamine. We can encode these evidences, and the links thereof, in an illustrative way, applying a directed acyclic graph (DAG). It is a useful method to identify and exemplify the concepts of exposure, outcome, causation, and confounding (16), especially when considering the complexities of CSA. DAG graphically sheds light on presumed relationships among variables, allowing bias adjustments in a standardized manner.

## Methods

### Participants

Our sample consisted of 70 outpatients (men and women, aged 15 to 66 years) experiencing TRD episode (unipolar and bipolar) referred by psychiatrists to the esketamine clinic in the Department of Psychiatry of the Federal University of São Paulo, Brazil, between April 2017 to December 2018. Eligibility criteria included: (a) TRD defined as the absence of response (<50% symptomatology reduction) in the current episode to at least two medication trials over an adequate period of time and with a minimal dose approved for the treatment; (b) the *Montgomery-Åsberg Depression Rating Scale* (MADRS) (17) severity score ≥25; and (c) stable physical health assessed by medical history. Exclusionary criteria were as follows: (a) history of hypersensitivity and/or allergy to esketamine; (b) diagnosis of esketamine abuse or dependence; (c) uncontrolled hypertension; or (d) pregnancy or breastfeeding.

### Measures

Each patient had one baseline assessment in which a certified psychiatrist conducted a structured clinical interview. This survey included socio-demographical questions and inquired about the medical history from the patients and their first-degree relatives, including the presence of alcohol and substances use disorders. A current major depressive episode diagnosis was confirmed applying the *Mini-International Neuropsychiatric Interview (MINI)-*plus 5.0 (18), and patient's history of childhood sexual trauma was assessed using the *Early Trauma Inventory Self Report-Short Form (ETISR-SF)* (19).

The ETISR-SF comprises six dichotomous (yes/no) questions about CSA posed in a steadily increasing emotional intensity whereby favoring its assessment (19). For this study, CSA is defined as yes responses to any of these six items. The scoring strategy is the simple method of counting the number of events that had occurred. The version translated and validated on Brazilian–Portuguese was applied. It was used in this study for being a brief (about 15 min) and feasible screening tool for quantitative and Qualitative childhood trauma in clinical practice. The Brazilian–Portuguese version has been shown to be suitable in terms of both validity and internal consistency for assessing traumatic experiences (alpha value for the total scale = 0.83 and alpha value for sexual events = 0.73; test–retest reliability = 0.78–0.90) (20).

The MADRS measured depression symptom severity at baseline and throughout the treatment.

### Subcutaneous Esketamine Protocol

**Figure 1:**
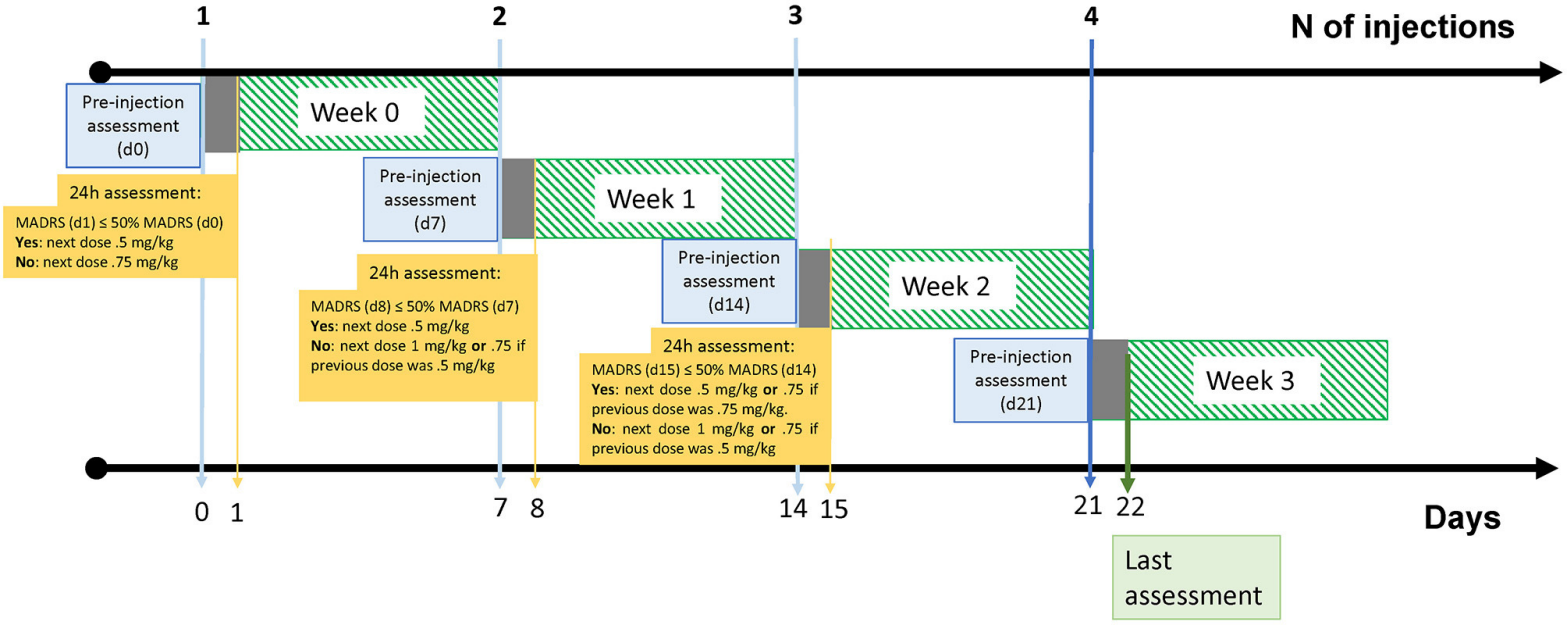
Graphical representation of the 4-week protocol. Days *0*, 7, *14* and *21* represents the days of dosing. Days *1, 8* and *15* correspond to 24 h post-injection MADRS assessment for dose titration. At the *day 0* in *week 0*, all patients received a.5 mg/kg dosing which could be augmented to.75 up to 1.0 mg/kg in the following weeks. *Day 22* in *week 3* represents the end of follow-up. Graphical design according to Schneeweiss, 2019 (21).

A titrating dose protocol with SC esketamine injection once a week for up to 4 weeks was conducted (Figure 1) (22). This protocol was administered by a certified psychiatrist with ACLS training and a nurse. Each patient had the same weekly schedule. Pre-injection ratings included the MADRS and vital signs (measurement of blood pressure, heart rate, and digital pulse oximetry). In the first session, all patients received 0.5 mg/kg of ketamine (ideal body weight) injected SC in the abdomen. We monitored vital signs at 15-min intervals for 120 min. A clinical assessment was performed before the patient was discharged, and they were accompanied by a close acquaintance.

Participants remained on their prescribed psychotropic medications, and all subjects provided written informed consent after a complete description of the protocol. This study was approved by the Federal University of São Paulo ethics committee.

### Statistical Analysis

#### Confounders Selection

Given the observational study design (i.e., non-experimental design), the relationship between exposure and outcome and potential confounders can be clarified using information from previous studies on the health consequences of CSA and predictors of response to depressive symptoms following treatments using ketamine; therefore, we conducted a search in the literature for retrieving those predictors. Then, we constructed a first DAG linking these predictors using the DAGitty graphical interface (23), in which the users promptly check changes in the diagram assessing the modifications of causal and biasing effects (24).

The main advantage in using this template is to select the confounders needed to adjust for (condition on) in the latent growth model to the extent that it would be possible to make valid inferences, answering the research question (25) based on findings that had been replicated in studies with greater samples. Finally, we removed the variables that were not in the path for confounding (i.e., covariates, which are not confounders *per se*), and created a summarized DAG for a clearer depiction. The medications currently prescribed for the participating patients (SSRI, SNRI, other classes of antidepressants, mood stabilizers, drugs for anxiety and insomnia, antipsychotics, and stimulants) were not employed as confounders.

#### Latent Growth Model Assessment

To estimate the effect of CSA, we fitted a latent growth model (26) using Mplus version 8.3 (27), where a latent intercept and a latent slope were estimated common to all individuals. The intercept is the systematic part of the variation in the outcome variable at baseline (i.e., time point zero) and slope growth factor is the trend, the growth rate, being the systematic part of the decrease (or increasing) in the outcome variable for a time score increase of one unit.

Latent growth model does not require complete data to estimate latent variables because under the assumption of missing at random mechanism, missingness is estimated unbiased via full-information maximum likelihood. Consequently, even when few patients had not responded to all evaluations, they were still considered in the analysis in an intention-to-treat approach given the maximum likelihood estimator.

Model fit was evaluated using the comparative fit index (CFI), root mean square error of approximation (RMSEA), and the standardized root mean square residual (SRMR). CFI values >0.95, RMSEA values of <0.06, and SRMR values >0.06, and a non-significant χ2 statistic were considered a good model fit (28).

## Results

### Sample Characteristics

Three patients were excluded from the initial sample (*n* = 70) because of missing 24-h post-injection data. The demographic and clinical characteristics of the 67 patients included are presented in Table 1.

**Table 1:** Clinical and demographic characteristics of patients (total *n* = 67) at the enrollment.

			**Unipolar depression**		**Bipolar depression**	
	***n*** **=** **67 (100%)**		***n*** **=** **37 (55.2%)**		***n*** **=** **30 (44.8%)**	
	**N/mean**	**(%/SD)**	**N/mean**	**(%/SD)**	**N/mean**	**(%/SD)**
CSA history	24	(35.8)	11	(29.7)	13	(43.3)
No-CSA history	43	(64.2)	26	(70.3)	17	(56.7)
Age of first episode (years)	21	(8.72)	22.76	(9.50)	19.00	(7.27)
BMI	29	(7.66)	27.26	(7.35)	31.19	(7.60)
Family history- SUD	22	(32.8)	11	(29.7)	11	(36.7)
MADRS baseline	30	(6.95)	33.19	(5.34)	34.00	(7.05)
Sex (female)	44	(65.7)	25	(67.6)	19	(63.3)
Age (years)	40	(12.93)	41.24	(13.23)	39.47	(12.70)
Educational level (college)	40	(59.70)	20	(54.1)	20	(66.7)
Employed	40	(59.70)	18	(48.6)	22	(73.3)

*CSA, child sexual abuse; N, number of patients; SD, standard deviation; BMI, body mass index; SUD, substance-use disorder*.

The prevalence of CSA among the 67 patients was 35.82%, with a sexual trauma load of 1 or higher (mean 1; SD: 1.45). Among patients reporting a CSA history, the proportions within the six ETISR-SF items were as follows: 31.3% disclosed a history of uncomfortable touching in intimate parts; 17.9% for having someone rubbing their genitals; 10.5% for being forced to touch genital parts; 7.5% for forced kiss; 7.5% for forced genital sex, and 4.5% for being forced to perform oral sex.

### First-Degree Relatives With Alcohol Use Disorder (+FH) and Sex as Confounders

**Figure 2:**
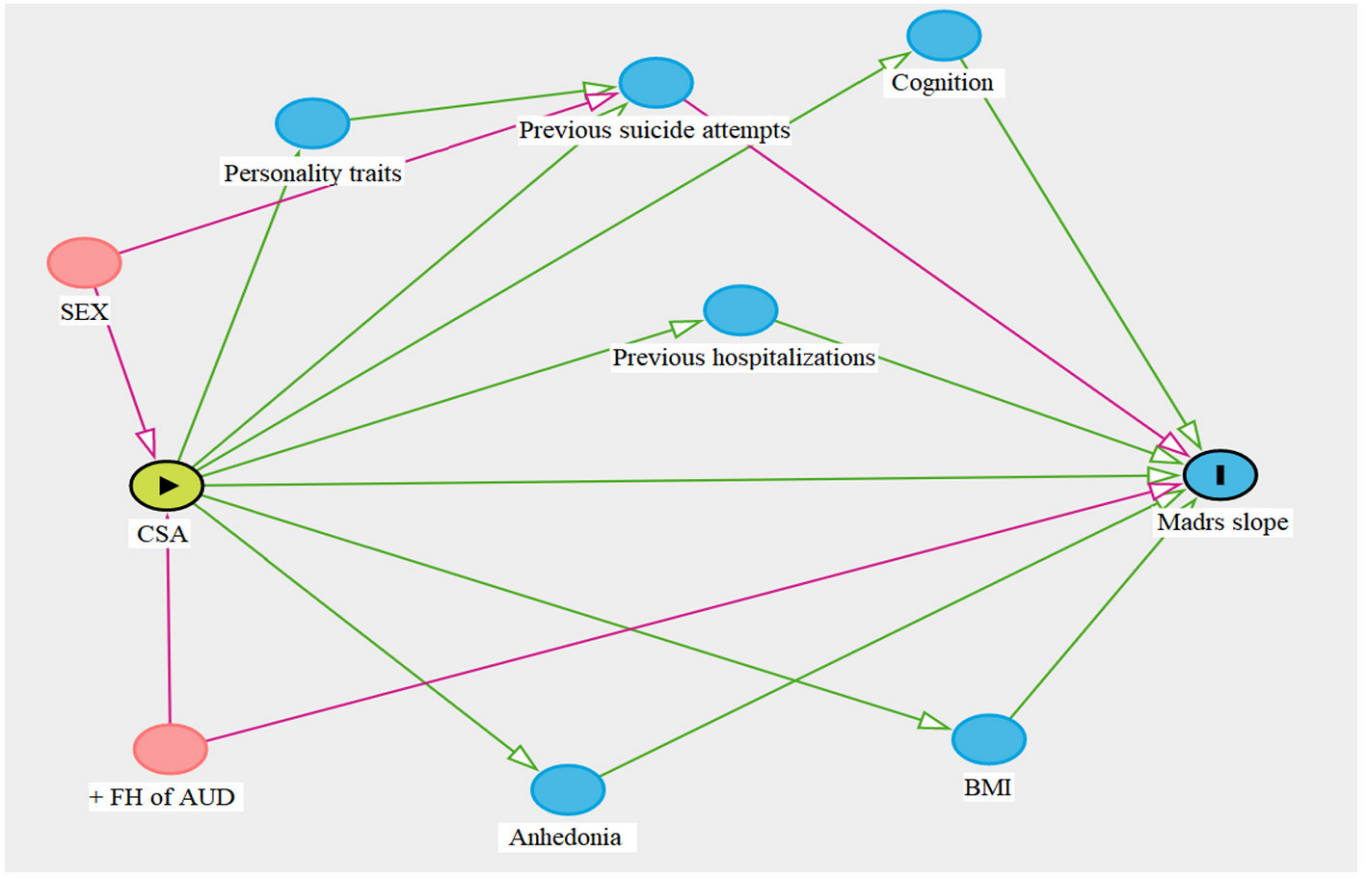
Summarized directed acyclic graphic (DAG). Previous studies were used to rule out the design process, during which the dagitty platform automatically generates red paths and nodes (sex and +FH of AUD), indicating which variables should be controlled in the statistical analysis; whereas green paths and blue nodes do not disrupt the interaction between exposure (▸) and outcome (■). Sex and +FH of AUD should be considered key moderating factors when investigating the effects of CSA on esketamine treatment. Personality traits, previous suicide attempts, hospitalizations, cognition, anhedonia, and BMI are predictors of treatment response, yet they do not disturb the relationship between the variables of interest. CSA, child sexual abuse; + FH AUD, positive family history of a first-degree relative with alcohol use disorder; BMI, body mass index.

The summarized DAG (Figure 2) showed that the presence of first-degree relatives with alcohol use disorder (+FH) and sex is a minimum set for adjustment (i.e., confounders) when analyzing the effects of esketamine in adults with CSA. These confounders were included in the latent growth model to minimize bias.

### Effect of Child Sex Abuse on Symptom Trajectory

For our main hypothesis, we observed that the overall presence of at least one CSA event was not a statistically significant predictor of the trajectory of symptom reduction [β (csa) = 0.386; *p* = 0.225; 95% CI = −0.137:0.909].

Table 2 displays mean MADRS scores according to the presence of CSA and depression subtype.

**Table 2:** Mean Montgomery–Åsberg depression rating scale (MADRS) score according to CSA history and depression subtype.

	**CSA history**							**No-CSA history**						
	**Unipolar**			**Bipolar**				**Unipolar**			**Bipolar**			
	**Mean**	**N**	**(SD)**	**Mean**	**N**	**(SD)**	***p***	**Mean**	**N**	**(SD)**	**Mean**	**N**	**(SD)**	***p***
Day 0	32.8	11	(1.5)	28.8	13	(2.5)	*0.190*	29.7	26	(1.2)	31.3	17	(1.7)	*0.453*
Day 1	17.8	9	(2.5)	20.9	13	(3.0)	*0.435*	19.6	23	(2.1)	23.3	16	(2.1)	*0.239*
Day 7	22.4	11	(2.8)	26.7	13	(2.5)	*0.265*	23.2	26	(1.8)	24.3	17	(2.6)	*0.200*
Day 8	16.8	10	(3.1)	20.6	12	(2.7)	*0.372*	18.2	22	(1.8)	22.8	17	(2.8)	*0.156*
Day 14	18.3	11	(3.1)	23.4	13	(3.7)	*0.318*	20.8	24	(1.7)	25.1	15	(2.9)	*0.180*
Day 15	14.7	9	(1.6)	19.9	11	(4.1)	*0.258*	17.7	22	(2.0)	17.2	12	(2.4)	*0.885*
Day21	23.1	11	(2.9)	20.7	12	(2.9)	*0.578*	19	25	(2.1)	22.8	17	(2.8)	*0.288*
Day22	16.4	8	(2.8)	15.2	8	(4.0)	*0.822*	14.3	24	(1.9)	18.2	16	(3.0)	*0.259*

*N, number of patients; SD, standard deviation. The italic values indicate the “p value”*.

## Discussion

To our knowledge, this is the first protocol investigating CSA as a clinical predictor of response of multiple subcutaneous esketamine injections in treatment-resistant depression.

In our model, +FH of alcohol use disorder and sex were highlighted as sources of bias between CSA and symptoms trajectory. Being raised by an alcohol-abusing parent increases the likelihood of sexual abuse exposure (29), and esketamine has been reported to be more effective in treatment-resistant depressed patients with a first-degree relative with alcohol use disorder (30). Child sexual abuse and +FH of alcohol use disorder are related to long-lasting neurodevelopmental or genetic/epigenetic variations (30) that predispose to depression in later life.

Female sex has a robust link to childhood sexual trauma and suicidal behaviors (2, 31). CSA is associated with a 2-fold increased risk for suicidal ideation, 3-fold risk for suicide attempts, and an 18-fold higher risk for dying by suicide (32). Conversely, esketamine has demonstrated rapid reductions in suicidal ideation (33). According to the diagrammed DAG, a way for clarifying the path among sex, suicidal ideation, and treatment outcome was controlling for sex as a confounder.

We hypothesized a negative effect of CSA on MADRS scores after repeated esketamine SC injections. However, even after adjustments for confounding factors (+FH of alcohol use disorder and sex), there was a lack of evidence of the effect of CSA and the esketamine response.

When investigating the effect of CSA on treatment response, it is essential to consider that abused patients have an increased probability of having suffered additional untoward experiences that may modify treatment response, disrupting data analysis. An advantageous method to avoid this not-randomizable limitation is by using a schematic representation for recognizing confounders. To this end, we first gathered knowledge from previous investigations, not constrained to our measured variables, thereby identifying common associations between CSA and outcome. Then, these relations were standardized in a DAG. This alternative approach allows a clearer perception of bias while measuring multiple potential confounders without creating other sources of distortions. It focuses on the understanding of prior empirical evidence between exposure and outcome, managing the selection of variables needed to be controlled in the statistical analysis (34). Hence, we could use a clear theoretical rationale to elect appropriate confounders, reducing bias and improving statistical data interpretation (35).

The outcomes from this naturalistic study suggests that, in contrast to the poorer response to conventional antidepressants, we have a lack of evidence that childhood sexual trauma results in a poorer response of depression symptoms to SC esketamine in the sample of patients participating in this study. Thus, the practical relevance of these findings is the need to screen for childhood trauma, detect patients that may not benefit from standard first-line antidepressants, and utilize adjunctive interventions, such as esketamine, especially for those with high suicide risk. Therefore, mental health providers should be conscious of the cumulative effect of CSA, assess child abuse not only as a hallmark of treatment-resistance but rather as a chronic treatment-resistant depression subtype.

Our study has several limitations, including the characteristics inherent to real-world analysis such as broad inclusion criteria, absence of a placebo-treated group, lack of randomization, small sample size, short length of follow-up, concomitant administration of different classes of psychotropic medications, and the usual scoring method of retrospective assessment of reported CSA in which detailed features of severity are not considered. Another limitation was the employment of the same protocol to unipolar and bipolar depression. However, Lucchese et al. (36) reported that there were no statistical associations between diagnosis and response to SC esketamine in this same sample (36). The results should be cautiously interpreted, and replication is essential.

The follow-up period of 4 weeks may have been too brief. As previously reported in an open-label trial of algorithm-guided pharmacotherapy, the rate of symptom improvement increases more slowly in patients with a history of childhood adversity. The difference in remission was more significant by the end of 12 weeks (32% compared with 44% of patients without abuse history) (13). The lack of early improvement at 2 to 4 weeks may be a predictor of later antidepressant non-response/non-remission (37). Therefore, we might theorize that the presence of CSA could slow even the rapid antidepressant response of esketamine, adding a potential need for treatment maintenance and more complexity in the care of this population.

## Data Availability Statement

The raw data supporting the conclusions of this article will be made available by the authors, without undue reservation.

## Ethics Statement

The studies involving human participants were reviewed and approved by Ethics committee of the Federal University of São Paulo. The patients/participants provided their written informed consent to participate in this study.

## Author Contributions

EM, LS, LD, ACL, CN, MT, CP, VR, RD, JS, MS, MB, GA, and AM: substantial contributions to the conception of the study and data acquisition. EM, LS, HC-M, and AM: analysis, or interpretation of data for the work. EM: drafting of the manuscript. LS, CBN, HC-M, JD, ALTL, and AM: critical review for important intellectual content. EM, LS, LD, ACL, CN, MT, CP, VR, RD, JS, MS, MB, GA, HC-M, JD, ALTL, and AM: final approval of the version to be published. All authors involved in this manuscript agree to be accountable for all aspects of the work, ensuring that questions related to the accuracy or integrity of any part of the work are appropriately investigated and resolved.

## Conflict of Interest

EM reports non-financial support from Torrent Pharma and non-financial support from Hypera Pharma, outside of the submitted work. LS reports personal fees from Daiichi Sankyo Brasil, Lundbeck Brasil, Pfizer, and Janssen, and non-financial support from Takeda Brasil, Moksha8 Brasil, Torrent Pharma, outside of the submitted work. CN reports non-financial support from Eurofarma, Cristália, and Sanofi, outside of the submitted work. Dr. Oliveira reports personal fees from Janssen outside of the submitted work. CBN research is supported by the NIMH grant MH-117293 and the National Institute on Alcohol Abuse and Alcoholism grant AA-024933. CBN has served as a consultant for Acadia Pharmaceuticals, Axsome, Compass Pathways, EMA Wellness, Epiodyne, Gerson Lehrman Group, Intra-Cellular Therapies, Janssen Research and Development, Magnolia CNS, Magstim, Navitor Pharmaceuticals, Signant Health, Sophos, Sunovion Pharmaceuticals, Taisho Pharmaceutical, Takeda, TC MSO, and Xhale, he is a stockholder in AbbVie, Antares, BI Gen Holdings, Celgene, Corcept Therapeutics Pharmaceuticals Company, EMA Wellness, OPKO Health, Seattle Genetics, TC MSO, Trends in Pharma Development, and Xhale, he has served on scientific advisory boards for the American Foundation for Suicide Prevention, the Anxiety Disorders Association of America (ADAA), the Brain and Behavior Research Foundation, the Laureate Institute for Brain Research, Magnolia CNS, Signant Health, Skyland Trail, and Xhale, he has served on boards of directors for ADAA, Gratitude America, and Xhale Smart, he has income sources or equity of $10,000 or more from American Psychiatric Association Publishing, CME Outfitters, EMA Wellness, Intra-Cellular Therapies, Magstim, Signant Health, and Xhale, and he has patents on a method and devices for transdermal delivery of lithium (US 6,375,990B1), on a method of assessing antidepressant drug therapy via transport inhibition of monoamine neurotransmitters by *ex vivo* assay (US 7,148,027B2), and on compounds, compositions, methods of synthesis, and methods of treatment (CRF receptor-binding ligand) (US 8,551,996B2). ALTL has received consulting fees from Janssen Pharmaceutical, Daiichi Sankyo Brasil, Cristalia Produtos Químicos e Farmacêuticos, Pfizer, Mantecorp Indústria Química e Farmacêutica, Libbs Farmacêutica, and Sanofi-Aventis over the last 24 months, and has received research fees from Janssen Pharmaceutical, Eli Lilly, H. Lundbeck A/S, Servier Laboratories, Hoffman-La Roche, and Forum Pharmaceuticals, not related to the submitted manuscript. AM has received non-financial support from Lundbeck not related to the present research. The remaining authors declare that the research was conducted in the absence of any commercial or financial relationships that could be construed as a potential conflict of interest.

## Publisher's Note

All claims expressed in this article are solely those of the authors and do not necessarily represent those of their affiliated organizations, or those of the publisher, the editors and the reviewers. Any product that may be evaluated in this article, or claim that may be made by its manufacturer, is not guaranteed or endorsed by the publisher.
