# Supplementary material for: A Clinical Rationale for Assessing the Impact of Childhood Sexual Abuse on Adjunctive Subcutaneous Esketamine for Treatment-Resistant Depression
Source: Front Psychiatry. 2021 Aug 17;12:608499. doi: 10.3389/fpsyt.2021.608499 (PMC8415867; doi:10.3389/fpsyt.2021.608499)
Supplement: Supplementary file 1 [file Data_Sheet_1.docx]

Supplementary Material

# Supplementary Figures and Tables

## Supplementary Figures


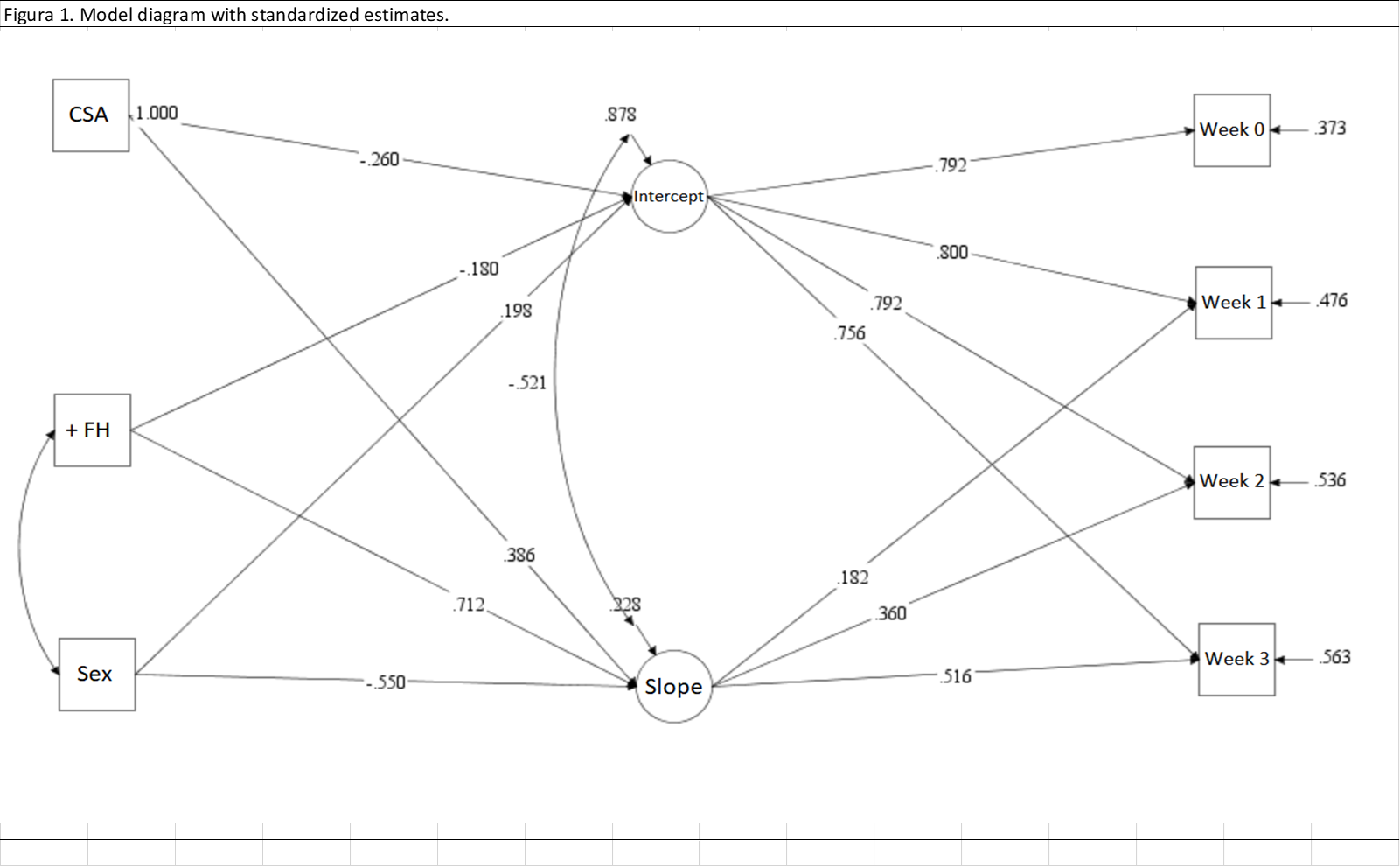


**Supplementary Figure 1.** Model diagram with standardized estimates.

**1.2 Supplementary Tables**

| Supplementary Table 1 │ Summary of analysis and model fit information | |
| --- | --- |
| Number of groups | 1 |
| Number of observations | 67 |
| Number of continuous latent variables | 2 |
| Number of Free Parameters | 17 |
| Loglikelihood | |
| H0 Value | -908.990 |
| H1 Value | -905.675 |
| Information Criteria | |
| Akaike (AIC) | 1.851.981 |
| Bayesian (BIC) | 1.889.461 |
| Sample-Size Adjusted BIC | 1.835.934 |
| Chi-Square Test of Model Fit | |
| Value | 6.632 |
| Degrees of Freedom | 13 |
| P-Value | 0.9201 |
| RMSEA | |
| Estimate | 0.000 |
| 90 Percent C.I | 0.000  0.043 |
| Probability RMSEA <= .05 | 0.958 |
| CFI/TLI | |
| CFI | 1.000 |
| TLI | 1.000 |
| Chi-Square Test of Model Fit for the Baseline Model | |
| Value | 68.115 |
| Degrees of Freedom | 18 |
| P-Value | 0.0000 |
| SRMR | |
| Value | 0.058 |

| Supplementary Table 2 │ Standardized model results. | | | | |
| --- | --- | --- | --- | --- |
|  | Estimate | Two-tailed p value | Confidence interval | |
|  |  |  | Lower 5% | Upper 5% |
| Intercept on CSA | -0.260 | 0.115 | -0.531 | 0.011 |
| Intercept on + FH of AUD | -0.180 | 0.233 | -0.428 | 0.068 |
| Intercept on Sex | 0.198 | 0.196 | -0.054 | 0.449 |
|  |  |  |  |  |
| Slope on CSA | 0.386 | 0.225 | -0.137 | 0.909 |
| Slope on AUD family-history | 0.712 | 0.087 | 0.027 | 1.397 |
| Slope on Sex | -0.550 | 0.135 | -1.156 | 0.056 |
| AUD, alcohol use disorder. | | | | |
